# Spatiotemporal Associations Between Ambient Air Pollution and Neoplasm Morbidity in Eastern Kazakhstan: Age-Specific Patterns and Spatial Heterogeneity, 2014–2024

**DOI:** 10.3390/ijerph23060785

**Published:** 2026-06-11

**Authors:** Gulnaz Sadykanova, Sanat Kumarbekuly, Ayauzhan Yessimbekova, Gulfat Kalelova

**Affiliations:** 1 Higher School of IT and Natural Sciences, S. Amanzholov University of East Kazakhstan, Ust-Kamenogorsk 070000, Kazakhstan; gsadykanova@vku.edu.kz (G.S.); gkalelova@vku.edu.kz (G.K.); 2 Faculty of Natural Sciences and Geography, Kazakh National Pedagogical University Named After Abay, Almaty 050010, Kazakhstan; 3 Branch “Hematology Department of East Kazakhstan Region” LLP “Center of Hematology”, Ust-Kamenogorsk 070010, Kazakhstan; ayauzhanyessimbekova@gmail.com

**Keywords:** ambient air pollution, environmental epidemiology, industrial region, neoplasm morbidity, East Kazakhstan, Spearman’s rank correlation

## Abstract

**Highlights:**

**Public health relevance—How does this work relate to a public health issue?**

- Neoplasm morbidity across the industrial settlements of the East Kazakhstan Region consistently exceeded the national average by a factor of 1.3 to 2.0 throughout 2014–2024, pointing to the presence of region-specific risk factors associated with the prevailing technogenic burden.
- Children and adolescents residing in areas of chronic atmospheric pollution in the vicinity of metallurgical and energy enterprises showed the most pronounced associations with gaseous pollutants (SO_2_, NO_2_, and CO), reflecting their heightened environmental vulnerability.

**Public health significance—Why is this work of significance to public health?**

- For the first time in the Central Asian context, this study simultaneously examines annual mean concentrations of four priority pollutants, the integrated API_5_ index, and age-stratified neoplasm morbidity across five industrial settlements over a ten-year observation period.
- Lag analysis revealed that reductions in industrial emission levels do not translate into immediate improvements in oncological indicators: in the adult population, statistically significant associations emerged at lag intervals of one to three years—a finding of critical importance for evaluating the effectiveness of environmental protection measures.

**Public health implications—What are the key implications or messages for practitioners, policy makers and/or researchers in public health?**

- Environmental regulatory authorities should introduce territorially differentiated emission controls for SO_2_ and NO_2_, with priority given to Ust-Kamenogorsk, where both the highest pollution levels and the strongest associations with morbidity among children and adolescents have been recorded.
- Healthcare systems in the industrial regions of Kazakhstan and Central Asia should incorporate environmentally vulnerable age groups—children aged 0–14 years and adolescents aged 15–17 years—into priority oncological screening programs and long-term epidemiological monitoring.

**Abstract:**

Industrial settlements of the East Kazakhstan Region face a persistent technogenic burden driven by the dense concentration of non-ferrous metallurgy and heat-and-power enterprises, further compounded by unfavorable pollutant dispersion conditions inherent to the region’s mountain–basin topography. This study evaluated spatiotemporal associations between annual mean concentrations of NO_2_, SO_2_, H_2_S, and CO, the integrated air pollution index (API_5_), and primary neoplasm morbidity across five settlements over the period 2014–2024. A retrospective ecological analysis was carried out for Ust-Kamenogorsk, Ridder, Altai, Shemonaikha, and the settlement of Glubokoe, incorporating Spearman’s rank correlation, lag analysis (1–3 years), and the Mann–Kendall trend test. Throughout the study period, neoplasm morbidity in the region consistently exceeded the national average by a factor of 1.3 to 2.0. In Ust-Kamenogorsk—where metallurgical SO_2_ and NO_2_ emissions are most heavily concentrated—strong positive associations were found in children for SO_2_ (*ρ* = 0.791, *p* < 0.05) and in adolescents for NO_2_ and CO, reflecting elevated inhalation exposure under conditions of chronic pollution. The negative associations with API_5_ observed in Ridder and Altai, where the index showed a statistically significant downward trend, are interpreted as evidence of the inertial character of oncological processes and the lasting influence of cumulative past exposure. Across all studied settlements, SO_2_ emerged as the most consistent predictor of morbidity variation. These findings support prioritizing stricter emission controls for SO_2_ and NO_2_ from metallurgical and energy facilities, establishing oncological screening programs for children and adolescents in chronically polluted areas, and strengthening ambient air monitoring—measures whose effective implementation will require coordinated action between public health authorities and environmental regulators.

## 1. Introduction

According to the World Health Organization, exposure to environmental factors, including ambient air pollution, is associated with millions of premature deaths worldwide each year [1,2]. Air pollution is recognized as one of the major environmental risk factors contributing not only to respiratory and cardiovascular diseases but also to the development of malignant neoplasms [3,4,5,6,7,8,9].

Two key indicators used throughout this study warrant brief clarification before proceeding. Primary neoplasm morbidity refers to the number of newly diagnosed tumors of all morphological types, both malignant and benign, registered for the first time and classified according to the ICD. This measure differs from incidence rates, which refer exclusively to malignant neoplasms. The Integrated Air Pollution Index for five priority substances (API_5_) is a composite indicator calculated as the weighted sum of the normalized annual mean concentrations of five priority pollutants, adjusted for their respective hazard classes. It characterizes long-term chronic atmospheric pollution and is used in Kazakhstani sanitary and hygienic practice as a generalized measure of ambient air quality. The full calculation formula is provided in the Methods section.

In 2013, the International Agency for Research on Cancer (IARC) classified ambient air pollution and particulate matter as Group 1 carcinogens, reflecting sufficient evidence of their role in lung carcinogenesis [10]. Since then, a substantial body of epidemiological, toxicological, and mechanistic evidence has confirmed the contribution of fine particulate matter (PM_2.5_) to carcinogenesis. Long-term exposure to PM_2.5_ has been linked to increased lung cancer mortality [11], while the meta-analysis by Hamra et al. [12] demonstrated a robust dose–response relationship between particulate concentrations and the risk of malignant neoplasms. Notably, similar associations have also been reported among never-smokers [13], highlighting the independent contribution of airborne exposures beyond tobacco smoke. Contemporary large-scale epidemiological studies likewise support the carcinogenic potential of ambient air pollution. In particular, a study published in Nature Communications demonstrated that prolonged exposure to PM_2.5_ was associated with an elevated risk of malignant neoplasms even at relatively low concentrations, pointing to the absence of a safe exposure threshold and emphasizing the importance of long-term environmental monitoring [14].

Recent studies have moved beyond the analysis of aggregate particulate concentrations and increasingly incorporate the chemical composition of aerosols. Spatiotemporal models based on remote sensing data and Bayesian exposure assessment approaches have revealed associations between the accumulation of organic carbon, sulfates, and dust fractions and the risk of lung cancer [15]. At the same time, increasing attention has been directed toward gaseous pollutants, including nitrogen dioxide (NO_2_), sulfur dioxide (SO_2_), and carbon monoxide (CO), which are involved in oxidative stress and inflammatory pathways that may promote carcinogenesis.

Multicomponent models indicate that concurrent exposure to particulate matter and gaseous pollutants may intensify adverse effects [16,17]. The absence of a safe threshold even at relatively low PM_2.5_ concentrations further highlights the significance of chronic exposure [18]. Accordingly, the contemporary risk assessment paradigm requires an assessment of the combined effects of multiple pollutants, with due consideration of their spatiotemporal dynamics.

Despite the substantial volume of international research, industrialized areas of Central Asia remain insufficiently represented in the scientific literature. Kazakhstan is characterized by a high concentration of metallurgical and power-generating enterprises in several industrial regions, which impose a persistent technogenic burden on the atmospheric environment. Studies conducted in various cities across the country have documented exceedances of the maximum permissible concentrations of priority pollutants and the presence of carcinogenic risks [19,20,21]. Accordingly, industrially developed regions with a high technogenic burden have become a particular focus of research on the health effects of environmental pollution. One such region is the East Kazakhstan Region, a major industrial center of Kazakhstan, where mining, non-ferrous metallurgy, and energy enterprises are highly concentrated [22]. The high concentration of industrial activity exerts substantial anthropogenic pressure on the environment. Environmental studies have demonstrated that ambient air in the region’s industrial cities contains a number of pollutants at concentrations exceeding the maximum permissible limits, including lead, sulfur dioxide, nitrogen oxides, phenol, and formaldehyde [22]. Ust-Kamenogorsk occupies a particularly important place among the region’s industrial centers, as it hosts major non-ferrous metallurgy and thermal power enterprises that constitute significant sources of atmospheric emissions [23]. According to studies assessing the environmental situation in Kazakhstan, the East Kazakhstan Region is among the country’s areas of pronounced environmental stress, owing to the combination of intensive industrial activity and a high concentration of technogenic pollution sources [24]. A quantitative relationship between total industrial emission volumes and neoplasm morbidity indicators in the East Kazakhstan Region was documented in our earlier work [25]. That analysis, however, had several important limitations. It was based on aggregated emission totals rather than annual mean concentrations of specific pollutants, and no composite air quality index was included. In addition, no age-stratified assessment was performed, meaning that children and adolescents, the most susceptible population groups, were not examined separately. Nor were lagged associations between changes in pollution levels and subsequent morbidity trends evaluated. To date, no published study in this region has simultaneously addressed all of the following: annual mean concentrations of priority gaseous pollutants (NO_2_, SO_2_, H_2_S, and CO) together with the integrated API_5_ index; age-disaggregated analysis of the affected population; and a decade-long observation period sufficient to detect delayed exposure effects. The present study was designed to fill this gap.

Against this background, examining the effects of ambient air pollution on population health indicators in this region is of substantial scientific and practical relevance. It should be emphasized that the present study uses the indicator of neoplasm morbidity, rather than focusing exclusively on malignant tumors. In this context, the term “neoplasms” is used in accordance with the International Classification of Diseases (ICD) and includes all newly registered tumor cases, covering both malignant and benign neoplasms.

The study was guided by the following hypotheses:

1. Age-related susceptibility. Due to their physiological characteristics and increased sensitivity to environmental exposures, children and adolescents may demonstrate stronger statistical associations between pollutant concentrations and morbidity indicators than adults.
2. Pollution–morbidity association. Statistical associations are expected between ambient air pollution levels and primary neoplasm morbidity rates. In this framework, the integrated air pollution index (API_5_) is considered a potentially more comprehensive indicator of the combined effects of multiple pollutants. Given the multifactorial nature of neoplasms, the present study evaluates associations exclusively at the population level.
3. Temporal lag effect. In cities with historically high but declining emission levels, such as Ridder and Altai, a temporal lag may exist between changes in ambient air quality and subsequent trends in morbidity indicators, reflecting the delayed impact of environmental exposures.

This study aimed to evaluate the spatiotemporal associations between annual mean concentrations of priority air pollutants (NO_2_, SO_2_, H_2_S, and CO), the integrated air pollution index (API_5_), and primary neoplasm morbidity rates across different age groups in industrially developed settlements of the East Kazakhstan Region during 2014–2024.

## 2. Materials and Methods

### 2.1. Study Area

The East Kazakhstan Region is situated in the eastern part of the Republic of Kazakhstan and represents one of the country’s major industrial regions.

The region has a population of approximately 724,000. Its spatial settlement structure is characterized by a high degree of urbanization, with a substantial share of the population concentrated in industrial centers. The largest city in the region is Ust-Kamenogorsk, which functions as its main industrial and administrative hub. Other important industrial centers include the cities of Ridder, Altai, and Shemonaikha, as well as the settlements of the Glubokoe District.

The regional economy is strongly industrialized. Non-ferrous metallurgy plays a dominant role and constitutes the backbone of industrial production in the region. Major metallurgical enterprises operating in the area include Kazzinc JSC, Ust-Kamenogorsk Titanium and Magnesium Plant JSC, and Ulba Metallurgical Plant JSC. Beyond metallurgy, the regional industrial structure also encompasses mechanical engineering, the energy sector, and processing industries.

**Figure 1:**
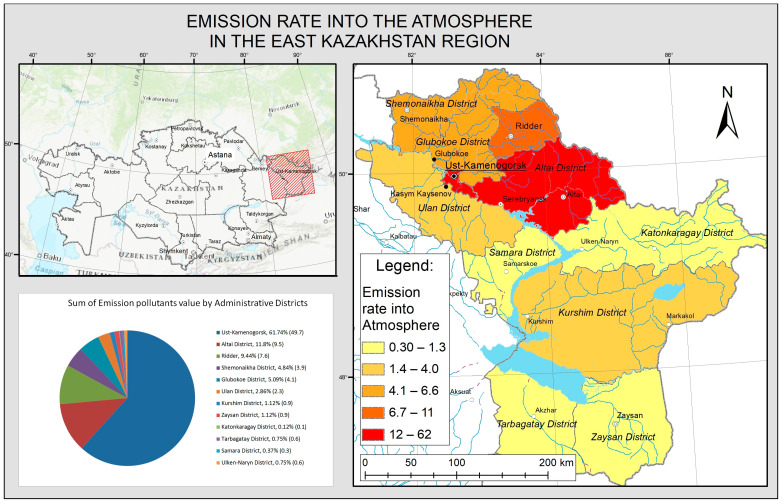
Geographic distribution of the studied industrial centers and the structure of atmospheric pollutant emissions across the administrative districts of the East Kazakhstan Region.

Industrial activity is spatially concentrated in the cities of Ust-Kamenogorsk, Ridder, Altai, and Shemonaikha, as well as in the Glubokoe District, resulting in the formation of territorially localized industrial zones (Figure 1).

### 2.2. Research Methods

The study was designed as a retrospective ecological analysis based on long-term observations of ambient air pollution indicators and neoplasm morbidity in the population of industrial areas of the East Kazakhstan Region during 2014–2024. The design was based on aggregated population-level data, in which the unit of observation was an administrative territory in a given calendar year (territory × year), thereby enabling the assessment of associations between environmental factors and health indicators at the population level.

The analysis encompassed the industrial settlements of the East Kazakhstan Region, namely Ust-Kamenogorsk, Ridder, Altai, Shemonaikha, and Glubokoe.

The observation period varied depending on data availability. For Ust-Kamenogorsk, Ridder, Altai, and the settlement of Glubokoe, the analysis covered an 11-year period (2014–2024), whereas for Shemonaikha it was limited to 5 years (2020–2024). Due to the small sample size for Shemonaikha, the resulting correlation coefficients (*ρ* = 1.0) were driven by the very limited number of observations (*n* = 5) and should not be interpreted as statistically robust estimates.

Ambient air quality monitoring is conducted within the framework of the state environmental surveillance system through a network of fixed monitoring stations located in the studied settlements. The source data were provided by the Republican State Enterprise Kazhydromet, the National Hydrometeorological Service of the Republic of Kazakhstan (https://www.kazhydromet.kz/, accessed on 27 November 2025). The monitoring system operates in accordance with national regulatory requirements and international air quality standards. The equipment and analytical methods employed are certified, and the quality assurance and quality control procedures comply with current standards.

Ambient air quality monitoring across the study area is conducted through a network of 17 observation posts distributed across five settlements in the East Kazakhstan Region. Ust-Kamenogorsk is served by 10 stations: five combined-type stations (manual sampling with automated capability) and five fully automated units. Ridder has three posts, including two combined-type stations and one fully automated unit. The settlement of Glubokoe has two posts: one manual sampling station and one automated unit. Altai and Shemonaikha are each served by a single fully automated station.

During database compilation, several isolated gaps were identified in the official Kazhydromet bulletins: CO data for Ridder in 2016; SO_2_, CO, and NO_2_ data for Altai in 2015; H_2_S data for the settlement of Glubokoe in 2014, 2023, and 2024; and H_2_S data for Shemonaikha in 2020. These omissions reflect the absence of the corresponding measurements in the official reporting records rather than any technical error in the database itself. Missing values were handled by mean imputation, whereby each absent observation was replaced with the arithmetic mean concentration of the respective pollutant for that settlement, calculated across all remaining years of the observation period. This approach was chosen because of the relative stability of industrial emissions in the region and the limited number of missing observations, which did not exceed three per settlement. It should nevertheless be acknowledged that mean imputation may reduce year-to-year variability and potentially underestimate the dispersion of the series; this limitation was taken into account when interpreting the correlation coefficients for the affected settlements.

Monitoring network density varies considerably across the study settlements. Ust-Kamenogorsk has the most extensive spatial coverage, with ten observation posts, whereas Altai and Shemonaikha each have only a single station. This limitation constrains intra-urban spatial analysis for these localities and was taken into account when interpreting the results.

The analysis covered four gaseous pollutants: H_2_S, SO_2_, CO, and NO_2_. Annual mean concentrations (mg/m^3^), derived from measurements collected at fixed Kazhydromet monitoring stations, were used throughout. Particulate matter (PM_2.5_ and PM_10_) was excluded from the correlation analysis for two reasons. First, these fractions are monitored only in Ust-Kamenogorsk (posts No. 2 and No. 3) and, to a limited extent, in Ridder (post No. 3 for PM_10_), whereas no corresponding data are available for Altai, Glubokoe, or Shemonaikha, thereby precluding meaningful cross-city comparisons. Second, the official annual bulletins published by Kazhydromet report maximum one-time concentrations for PM_2.5_ and PM_10_, whereas correlation analysis requires settlement-specific annual mean values, which are not publicly available.

For the integrated assessment of air pollution levels, the air pollution index (API_5_) was applied, being calculated from the normalized concentrations of priority pollutants as follows:

(1) $${API}_{i}=\sum_{i=1}^{n}{\left(\frac{q_{cp.i}}{{MPC}_{c.ci}}\right)}^{C_{i}}$$

where *q_cp.i._* denotes the average concentration of the *ith* pollutant, *MPC_c.ci_* denotes the maximum permissible daily average concentration of the *ith* pollutant, and *C_i_* is a coefficient accounting for the pollutant’s hazard class.

In addition to the *API_5_* index, the analysis incorporated two supplementary air quality indicators used within Kazakhstan’s sanitary and hygienic regulatory framework.

The Standard Index (SI) is defined as the ratio of the highest one-time concentration of a given pollutant recorded over the analysis period to its maximum permissible short-term concentration (*MPC_s.t._*):

*SI = C_max_/MPC_s.t._* (2)

where *C_max_* is the highest measured one-time concentration (mg/m^3^), and *MPC_s.t._* is the maximum permissible short-term concentration (mg/m^3^). The *SI* reflects the intensity of short-term peak pollution episodes.

The Highest Exceedance Frequency (HEF) is the proportion, expressed as a percentage, of single-measurement samples in which the MPC*_s.t._* was exceeded relative to the total number of samples collected over the analysis period:

*HEF* = (*n_exceed_*/*N_total_*) × 100% (3)

where *n_exceed_* is the number of samples in which the *MPC_s.t._* was exceeded, and *N_total_* is the total number of samples collected. The *HEF* reflects how frequently the population is exposed to pollutant concentrations above the regulatory threshold.

The combined use of SI and HEF makes it possible to distinguish between situations characterized by infrequent high peak concentrations and those marked by moderate but systematic exceedances of regulatory thresholds. Together with the API_5_ index, which captures chronic cumulative exposure, these indicators provide a comprehensive characterization of ambient air pollution conditions.

The degree of ambient air pollution was assessed using the classification scale presented in Table 1.

**Figure 2:**
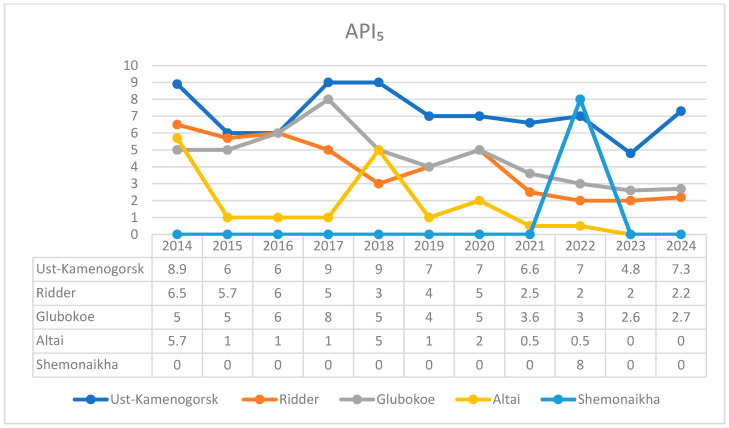
Temporal dynamics of the air pollution index (API_5_) across the industrial areas of the East Kazakhstan Region.

The zero API_5_ values shown in Figure 2 (for Altai in 2023–2024 and for Shemonaikha in certain years) reflect the actual values documented in the official Kazhydromet reporting records rather than missing observations.

In addition, two supplementary pollution indicators were considered: the standard index (SI), calculated as the ratio of the maximum one-time concentration to the maximum permissible concentration, and the highest frequency of exceedance (HFE), which characterizes the proportion of observations in which the maximum permissible concentration was exceeded.

The dependent variable was primary neoplasm morbidity (per 100,000 population), classified in accordance with the International Classification of Diseases (ICD). The data were provided by the East Kazakhstan Regional Branch of the Republican State Enterprise Salidat Kairbekova National Research Center for Health Development under the Ministry of Health of the Republic of Kazakhstan and were derived from the official medical registration system.

In the present study, the indicator “neoplasms” is used as an aggregated epidemiological measure that encompasses different tumor types regardless of their histological structure.

The database was compiled for the period 2014–2024 and included indicators for the following population groups:

- the total population;
- adults (≥18 years);
- adolescents (15–17 years);
- children (0–14 years).

Age stratification was applied to identify differences in the distribution of morbidity across demographic groups.

Statistical analysis was conducted using Statistica, version 13.0 (TIBCO Software Inc., Palo Alto, CA, USA). To evaluate the associations between ambient air pollutant concentrations and neoplasm morbidity indicators, Spearman’s rank correlation coefficient (*ρ*) was applied, owing to the limited sample size, the potential non-normality of the data distribution, and the presence of outliers.

(4) $$\rho =1-\frac{6{\sum d}_{i}^{2}}{n(n^{2}-1)}.$$

where d*_i_* denotes the difference between ranks, and *n* denotes the number of observations.

The use of a nonparametric approach partially reduces the influence of uncontrolled factors, including smoking, climatic and meteorological conditions, and infectious morbidity.

To assess the potential delayed effects of ambient air pollution, lagged analyses were conducted using 1-, 2-, and 3-year lag periods. Associations were evaluated separately for each settlement and age group.

To statistically confirm spatial differences among settlements with respect to SI and HEF, the nonparametric Kruskal–Wallis test was applied, as it does not assume a normal distribution. Pairwise comparisons were performed using the Mann–Whitney test with Bonferroni correction for multiple comparisons (adjusted significance threshold: *α* = 0.005). Descriptive statistics are presented as the mean, standard deviation (SD), median, and 95% confidence interval.

To assess the direction and statistical significance of temporal trends in the API_5_ index, the nonparametric Mann–Kendall test was applied, and the magnitude of the trend slope was estimated using Sen’s slope method.

Statistical significance was defined at *p* < 0.05. The study was based on aggregated data and did not account for individual-level risk factors, including smoking, occupational exposures, and genetic susceptibility.

ArcGIS Pro software version 3.2 was used to create cartographic materials.

## 3. Results

### 3.1. Air Quality Data and Environmental Monitoring

Analysis of integrated ambient air pollution indicators revealed pronounced spatial heterogeneity in the technogenic burden across the study region (Table 2).

Table 2 highlights the clear dominance of Ust-Kamenogorsk in terms of peak pollution intensity, whereas the remaining settlements exhibit less severe pollution profiles with varying exceedance frequencies. According to both indicators, Ust-Kamenogorsk falls within Grade IV, corresponding to a “very high” level of pollution. In terms of maximum SI values, Ust-Kamenogorsk exceeded the other settlements by approximately 15- to 26-fold; however, differences in annual mean values were less pronounced, as confirmed by the descriptive statistics and pairwise comparison results. Ridder and Glubokoe are classified within Grade III, corresponding to a “high” level of pollution. Altai and Shemonaikha also fall within Grade III on the basis of their SI values, although their HEF values are considerably lower, indicating a less systematic pattern of threshold exceedance in these localities.

The Kruskal–Wallis test revealed statistically significant inter-settlement differences in the Standard Index (SI) (*p* < 0.001), whereas no significant differences were found for HEF (*p* = 0.292). Pairwise comparisons showed that Ust-Kamenogorsk differed significantly from Ridder, Glubokoe, and Altai in terms of the SI, while no statistically significant differences were detected among the remaining settlements (Table 3). The high standard deviation of the SI in Ust-Kamenogorsk reflects pronounced year-to-year variability driven by extreme pollution episodes in certain years. The absence of significant differences in HEF indicates that the frequency of MPC*_s.t._* exceedances is broadly comparable across the study settlements. Thus, Ust-Kamenogorsk stands out not in how often threshold exceedances occur but in the intensity of the peak pollution episodes when they do.

Ust-Kamenogorsk thus emerges as the unequivocal regional leader in atmospheric pollution intensity, with critical values recorded for both peak concentrations and exceedance frequency. The remaining settlements exhibit high, although less systematic, levels of pollution.

Temporal trends in the Air Pollution Index (API_5_), a composite indicator of chronic pollution exposure, over the period 2014–2024 revealed pronounced spatial heterogeneity across the study area (Figure 2).

Throughout the entire observation period, Ust-Kamenogorsk maintained a persistently high level of pollution, never falling below the “high” classification in any of the years analyzed. In Ridder and Glubokoe, a downward trend in API_5_ was observed, with values declining by 66% and 46%, respectively, over the observation period (Figure 2).

In Altai, API_5_ values declined to zero in 2023–2024 (Figure 2). This finding should be interpreted with caution: although the zero values reflect the official Kazhydromet reporting data, they may partly result from changes in the set of monitored pollutants or from reduced monitoring coverage rather than from an actual absence of pollution. Shemonaikha was characterized by high year-to-year variability, with a sharp increase in API_5_ to 8.0 in 2022 against a background of zero values in the remaining years (Figure 2), most likely reflecting the episodic nature of the technogenic burden in that locality.

Thus, over the 2014–2024 period, two contrasting pollution trajectories emerged across the region: persistent chronic pollution in Ust-Kamenogorsk, on the one hand, and a statistically confirmed decline in API_5_ in Ridder, Glubokoe, and Altai, on the other, albeit with remaining uncertainty as to whether the improvements recorded in Altai reflect genuine emission reductions or changes in monitoring practice.

The Mann–Kendall test confirmed statistically significant downward trends in API_5_ for Ridder, Glubokoe, and Altai (*p* < 0.05), whereas no significant trends were detected for Ust-Kamenogorsk or Shemonaikha (Table 4). The steepest decline was observed in Ridder (Sen’s slope = −0.50 units/year), consistent with a sustained long-term improvement in air quality in that settlement.

The statistical trend analysis confirms that the observed decline in API_5_ across three of the five settlements represents a consistent pattern rather than random fluctuation, thereby providing a foundation for the subsequent examination of associations between pollution dynamics and population morbidity indicators.

The studied settlements thus fall into three distinct groups based on their pollution profiles. The first group comprises Ust-Kamenogorsk, where pollution levels remained persistently high and reached the “very high” classification in certain years. The second group includes Ridder and Glubokoe, where an initially high level of pollution was accompanied by a statistically significant downward trend in API_5_. The third group encompasses Altai and Shemonaikha, characterized by predominantly moderate pollution levels and episodic threshold exceedances.

The decline in API_5_ observed in Ridder, Glubokoe, and Altai may not be attributable solely to actual reductions in emissions. Changes in industrial activity, modernization of individual production processes, shifts in the composition of monitored pollutants, and features of the monitoring network may all have contributed to this trend. The observed changes should therefore be interpreted as a statistically confirmed shift in the composite air quality indicator, rather than as direct evidence of the complete elimination of the technogenic burden.

### 3.2. Spatiotemporal Dynamics of Neoplasm Morbidity

Neoplasm morbidity rates in the East Kazakhstan Region consistently exceeded the national average by a factor of 1.3 to 2.0 throughout the entire study period (Table 5). The highest excess ratio was recorded in 2014–2015, when the region ranked second to third in the national standings. The sharp drop in case detection during 2020–2021 reflects diagnostic constraints imposed by the COVID-19 pandemic rather than any genuine improvement in the epidemiological situation. In the subsequent years, morbidity indicators recovered to pre-pandemic levels.

Throughout the entire observation period, the East Kazakhstan Region thus exhibited a persistently elevated level of neoplasm morbidity relative to the national average—a pattern that points to the existence of region-specific risk factors and provides grounds for examining their association with ambient air pollution indicators.

### 3.3. Correlation Analysis of Air-Health Associations

**Figure 3:**
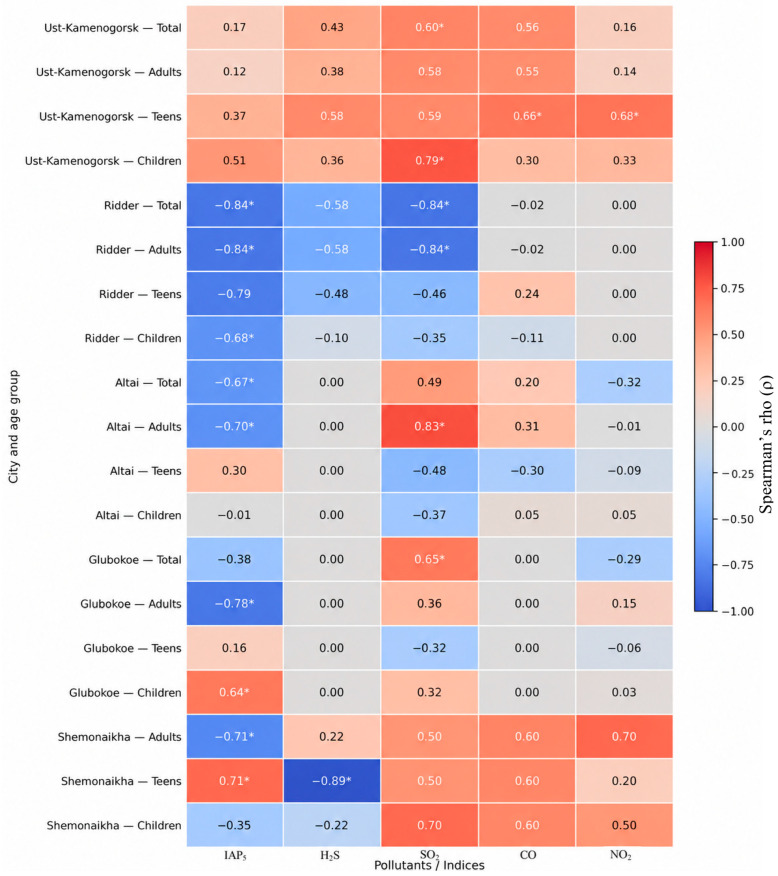
Heat map of Spearman’s rank correlation coefficients between ambient air pollutant concentrations and neoplasm morbidity by city and age group (2014–2024, *p* < 0.05). Note: * *p* < 0.05. Spearman’s correlation coefficient (ρ) was calculated for each pollutant–population group pair. Positive values (red) indicate a direct association, while negative values (blue) indicate an inverse association.

Spearman’s rank correlation analysis revealed territorially and age-specifically differentiated associations between ambient air pollution and neoplasm morbidity (Figure 3).

In Ust-Kamenogorsk, the most pronounced positive associations were found in the pediatric population: with SO_2_ (*ρ* = 0.791, *p* < 0.05) and API_5_ (*ρ* = 0.507). Among adolescents, the strongest associations were identified for NO_2_ (*ρ* = 0.679) and CO (*ρ* = 0.661). These findings point to heightened sensitivity of the younger age groups to gaseous pollutants.

Ridder and Altai: consistent negative correlations were found between API_5_ and morbidity in the adult population (*ρ* = −0.922 and −0.701, respectively). Notably, in Ridder SO_2_ showed a strong negative correlation (*ρ* = −0.845), whereas in Altai the same indicator displayed a strong positive association (*ρ* = 0.826)—the opposing directions of these associations in two cities sharing a similar pollution decline profile suggest a possible lag effect between changes in exposure and subsequent morbidity trends.

Glubokoe: a statistically significant positive correlation was found between SO_2_ and overall morbidity (*ρ* = 0.654, *p* < 0.05).

Shemonaikha: the results (*n* = 5) cannot be regarded as statistically reliable and are presented solely as preliminary observations.

The spatial structure of the identified associations is visualized in the heat map (Figure 3).

The most consistent positive associations were thus identified in Ust-Kamenogorsk—a city with chronically high pollution levels—predominantly among children and adolescents. The opposing directions of correlations observed in Ridder and Altai call for further analysis incorporating lag effects and pollution dynamics, which is addressed in the following subsection.

### 3.4. Lag-Dependent Associations Between Air Pollution and Neoplasm Incidence

Lag analysis revealed pronounced spatial and age-specific heterogeneity in the associations between ambient air pollution and neoplasm morbidity (Table 6).

A key pattern emerging from this analysis is that statistically significant associations in children were predominantly observed at lag 0, whereas in adults they tended to appear at lags of one to three years. This may reflect a higher immediate reactivity of the child organism alongside a comparably longer latency period in the adult population.

SO_2_ emerged as the most consistent predictor across all settlements: statistically significant associations with this pollutant were identified in Ust-Kamenogorsk, Ridder, Altai, and Glubokoe across different age groups and at varying lag intervals. The integrated API_5_ index showed significant associations predominantly at lags of one to three years, which is consistent with its nature as a measure of chronic cumulative exposure.

The results for Shemonaikha (*ρ* = 1.00) are an artifact of the extremely small number of observations (*n* = 5) and cannot be regarded as statistically reliable.

The lag analysis thus supports the hypothesis of a delayed effect of ambient air pollution on neoplasm morbidity, most pronounced in the adult population of cities where pollution levels have been declining—namely Ridder and Altai.

## 4. Discussion

The identified spatiotemporal and age-specific differences play an important role in shaping the observed associations between ambient air pollution and neoplasm morbidity in industrial regions. The most consistent and statistically significant relationships identified in the present study were observed among children and adolescents, indicating that these groups may serve as the most sensitive indicators of adverse environmental conditions.

In Ust-Kamenogorsk, a strong positive correlation was observed between sulfur dioxide (SO_2_) concentration and neoplasm morbidity in children (*ρ* = 0.791; *p* < 0.05). This association may be attributable to physiological characteristics of children, including a higher respiratory rate and a greater relative alveolar surface area, both of which may increase inhalation exposure. In the adolescent group, statistically significant correlations were identified for NO_2_ and CO, suggesting a heightened sensitivity of this age group to ambient air pollutants.

The heightened susceptibility of children is consistent with findings from a cohort study conducted in the Netherlands under the leadership of M. Guxens (Erasmus University, Rotterdam), which demonstrated that prenatal exposure to air pollution is associated with alterations in brain morphology and reduced cognitive performance in children [26]. These findings suggest that the effects of pollutant exposure may emerge as early as the initial stages of ontogenesis and may carry long-term implications. The age-related differences in the pattern of associations reflect not only physiological characteristics but also differences in exposure patterns. Children spend a substantial proportion of their time outdoors in close proximity to their place of residence, which increases their cumulative inhalation burden. Furthermore, the immaturity of the child’s immune and antioxidant systems reduces the organism’s capacity to neutralize the genotoxic effects of pollutants, potentially contributing to a more rapid manifestation of pathological changes compared to adults.

The findings also highlight the importance of multicomponent pollutant exposure. The positive correlation between the integrated air pollution index (API_5_) and morbidity among children and adolescents in Ust-Kamenogorsk, together with the associations observed in Shemonaikha despite the absence of pronounced relationships for individual pollutants, may suggest that combined exposure to pollutant mixtures plays a more prominent role than the isolated effects of single components.

Comparable conclusions were reached in the study by Z. Khorrami (Tehran University of Medical Sciences), which demonstrated that combined exposure to multiple pollutants, including BTEX and NOx, showed stronger associations with lung cancer morbidity than individual pollutants analyzed separately [27]. This suggests the possibility of a synergistic effect arising from simultaneous exposure to multiple air contaminants. Similar evidence was reported by M. F. Gabriel et al. (Portugal), who showed that children are exposed to complex mixtures of volatile organic compounds across different microenvironments, and that it is the cumulative exposure that reflects a persistent technogenic burden [28]. The findings of the present study are in line with this concept.

Ecological studies from different countries likewise support the patterns identified in the present study. In the United States, L. C. Vinikoor-Imler (University of North Carolina) demonstrated a positive association between PM_2.5_ concentrations and lung cancer morbidity at the population level [29]. In Taiwan, S.-Y. Su (National Taiwan University) reported a statistically significant relationship between PM_2.5_ and cancer morbidity, mediated through mechanisms of oxidative stress, inflammation, and DNA damage [30]. In Japan, K. Hasegawa (University of Occupational and Environmental Health) identified dose–response relationships between NO_2_, SO_2_, PM_2.5_, and various cancer types [31]. Despite differences in study design, all of these studies support the role of air pollution as a significant carcinogenic factor.

From a mechanistic perspective, the present findings are consistent with experimental and clinical studies conducted in Kazakhstan. Exposure to industrial pollution has been shown to induce pronounced biochemical alterations, including increased activity of liver enzymes (ALT, AST, and GGT), disturbances in carbohydrate and protein metabolism, and the development of tissue hypoxia [32,33]. These processes reflect the activation of oxidative stress, inflammatory responses, and cellular damage, which are widely regarded as key mechanisms underlying carcinogenesis.

Further evidence for the role of multicomponent pollution was reported by F. Dominici (Harvard T.H. Chan School of Public Health, USA), who demonstrated that not only the mass concentration of PM_2.5_ but also its chemical composition—particularly sulfate content—is associated with differences in health effects and life expectancy [34]. This indicates that individual pollution components may exert unequal and potentially divergent effects, thereby contributing to complex and non-linear associations.

The negative correlations observed in Ridder and Altai (up to *ρ* = −0.922) point to the complex nature of environmental associations and may be attributable to a temporal lag between changes in pollution levels and subsequent morbidity trends, although this hypothesis requires further verification. As noted by Feng Y. [35], the effects of pollutant exposure may emerge with a delay. Moreover, according to F.W. Lipfert and R.E. Wyzga, the latency period for cancer development may extend over several decades [36]. Accordingly, current morbidity patterns may partly reflect pollution exposures that occurred in earlier periods.

It is equally important to acknowledge that despite officially reported reductions in industrial emission volumes across the region, actual ambient air quality may remain unsatisfactory. Assanov et al. [23] demonstrated that in Ust-Kamenogorsk, concentrations of NO_2_ and SO_2_ consistently exceed regulatory thresholds throughout the year regardless of season, and that declining official emission statistics are not accompanied by measurable improvements in air quality. This implies that the negative correlations between current API_5_ levels and morbidity in Ridder and Altai may reflect not a genuine improvement in the environmental situation, but rather a temporal lag between historically high cumulative exposure and the eventual clinical manifestation of oncological pathology.

Large-scale epidemiological studies further support the substantial impact of air pollution on human health. In a multicenter study encompassing 652 cities in 24 countries, C. Liu (China CDC) demonstrated that even short-term increases in PM_2.5_ concentrations were associated with elevated mortality [37]. Long-term effects were likewise confirmed in a European cohort study led by R. So (International Agency for Research on Cancer), which identified an association between NO_2_ exposure and the risk of liver cancer [38].

Regional evidence likewise supports the present findings. In the East Kazakhstan Region, the high concentration of mining and metallurgical enterprises creates a persistent technogenic burden, accompanied by unfavorable population health indicators [39]. In Ust-Kamenogorsk, ambient air pollution levels have remained consistently high in recent years as a result of industrial emissions [40], thereby reinforcing the relevance of the associations identified in the present study.

The physical-geographical and meteorological characteristics of the region may amplify the observed associations. The combination of mountainous terrain and unfavorable meteorological conditions, including temperature inversions and periods of low wind speed, restricts pollutant dispersion and promotes their accumulation in the near-surface layer of the atmosphere. This may increase population exposure levels, particularly in industrial centers such as Ust-Kamenogorsk and Ridder, where more pronounced associations have been observed between gaseous pollutant concentrations and morbidity indicators.

It should be noted that the observed correlation coefficients were predominantly moderate in magnitude, reflecting the multifactorial nature of oncological diseases. The effects of air pollution are likely to act in combination with other risk factors, including smoking, occupational exposures, and socioeconomic conditions, as supported by ecological studies [27,29,31].

Assanov, Zapasnyi, and Kerimray [41] found that the majority of large industrial enterprises in Kazakhstan actually increased—rather than reduced—their permitted emission volumes during the most recent licensing period, while Kazakhstan’s air quality standards for SO_2_ are 25 times less stringent than WHO guidelines. Against this backdrop, the associations identified in the present study between pollution levels and neoplasm morbidity in children and adolescents underscore the need not only to tighten permitting standards for industrial facilities in the region, but also to harmonize national air quality regulations with international WHO benchmarks.

The findings carry implications for environmental policy and public health. They demonstrate that uniform regional approaches to air quality management are insufficient for industrial territories with differing pollution profiles. For Ust-Kamenogorsk, the priority should be controlling peak pollution episodes and emissions from the largest industrial enterprises. For settlements where API_5_ has been declining, sustained monitoring is needed to distinguish genuine environmental improvement from changes in the composition of monitored pollutants or in monitoring coverage. The more pronounced associations observed among children and adolescents justify targeted preventive measures in schools, residential areas, and other spaces where children spend extended periods of time.

Taken together, the findings highlight the importance of accounting for cumulative pollutant exposure, age-related population susceptibility, and the spatial heterogeneity of environmental factors when developing regional strategies for ambient air monitoring and cancer prevention.

Promising directions for future research include cohort studies incorporating individual-level exposure assessment and lifestyle factors. Extending the analysis to respiratory and cardiovascular diseases would also be worthwhile. The inclusion of data on the chemical composition of PM_2.5_ and PM_10_ represents a further important avenue. For Ust-Kamenogorsk, which benefits from the most developed monitoring network in the region, the application of spatial models to assess intra-urban exposure heterogeneity offers particular promise.

## 5. Conclusions

The present study offers the first comprehensive spatiotemporal analysis of associations between annual mean concentrations of priority gaseous pollutants (NO_2_, SO_2_, H_2_S, and CO), the integrated API_5_ index, and primary neoplasm morbidity with age-stratified population data across five industrial settlements of the East Kazakhstan Region over the ten-year period 2014–2024. Unlike previous work conducted in this region, this study simultaneously accounts for multiple pollutants, disaggregates the population by age group—with particular attention to children and adolescents as the most vulnerable subpopulation—and evaluates lag effects, thereby substantially expanding the evidence base for this geographical context.

The central finding of the study is the demonstrated age-related vulnerability: the most pronounced and statistically significant associations were identified among children and adolescents, indicating their heightened susceptibility to the technogenic burden and providing a strong rationale for prioritizing the protection of these population groups. Sulfur dioxide emerged as the most consistent predictor across all settlements, while the integrated API_5_ index showed significant associations predominantly at lags of one to three years, consistent with the chronic nature of its exposure effect. The spatial heterogeneity of pollution was statistically confirmed: Ust-Kamenogorsk consistently stands out as a territory with very high peak ambient air pollution levels, whereas Ridder and Glubokoe show a statistically significant decline in API_5_.

These findings support the need to prioritize ambient air quality control in the vicinity of educational institutions, residential areas, and other spaces where children spend extended periods; to revise permitting standards for industrial enterprises; and to establish an integrated long-term population health monitoring system in the industrial regions of Kazakhstan. Given the ecological study design and limited sample size, the results should be regarded as a foundation for future cohort studies that incorporate individual-level risk factors.

### 5.1. Limitations of the Study

Despite the significance of the findings, the study carries several limitations. The use of aggregated population-level data cannot preclude the ecological fallacy, as city-wide mean pollutant concentrations may not reflect individual exposure doses. The heterogeneity of the time series—five years for Shemonaikha versus eleven years for the remaining settlements—constrains the statistical power of inter-city comparisons, and the application of mean imputation to address isolated gaps in the Kazhydromet data may attenuate year-to-year variability and underestimate series dispersion. The range of monitored pollutants differs substantially between settlements—from two indicators in Altai to twenty in Ust-Kamenogorsk—which limits the scope for meaningful cross-city comparison. Particulate matter fractions PM_2.5_ and PM_10_ were excluded from the analysis due to the absence of annual mean concentrations disaggregated by settlement within the Kazhydromet monitoring system. Finally, the study does not account for individual-level risk factors—including smoking, occupational exposures, genetic predisposition, and socioeconomic conditions—whose contribution to cancer morbidity is well established. The population-level patterns identified here should be regarded as a basis for subsequent cohort studies conducted with due consideration of these limitations.

### 5.2. Use of Artificial Intelligence

Artificial intelligence tools were used exclusively for grammatical, stylistic, and structural editing of the manuscript. They were not used to generate scientific data, conduct analyses, interpret the findings, or draw the study’s conclusions. Full responsibility for the analyses, interpretation of results, and scientific content of this manuscript rests with the authors.

## Figures and Tables

**Table 1:** Assessment of Air Pollution Index (API_5_) Levels.

Grade	Air Pollution Level	Indicators	
I	Low	0–1	0
II	Elevated	2–4	1–19
III	High	5–10	20–49
IV	Very High	>10	>50

Note: This classification scale is used for the monthly and annual assessment of ambient air pollution levels in accordance with the current regulatory documents of the Republic of Kazakhstan.

**Table 2:** Air Pollution Indicators and Major Pollutants in Industrial Settlements of the East Kazakhstan Region.

Industrial Settlement	Maximum Exceedance Factor Relative to the MPC_d.a._	Maximum SI	Maximum HFE (%)	Major Pollutants
Ust-Kamenogorsk	up to 2.4 × MPC	132	84	H_2_S, SO_2_, NO_2_, CO
Ridder	up to 1.3 × MPC	8.6	69	H_2_S, SO_2_
Glubokoe	up to 1.7 × MPC	9	33	SO_2_, H_2_S, NO_2_
Altai	<1 × MPC	5	44	NO_2_, CO
Shemonaikha	up to 3.0 × MPC	6	15	NO_2_, SO_2_

Note: MPC_d.a._ = maximum permissible daily average concentration; SI = standard air pollution index; HFE = highest frequency of exceedance of the maximum one-time permissible concentration.

**Table 3:** Descriptive Statistics for SI and HEF by Settlement, 2014–2024.

Industrial Settlement	*n*	Mean SI	SD	Median SI	95% CI for the Mean SI	Mean HEF (%)	SD
Ust-Kamenogorsk	11	25.63	38.05	9.70	[3.14; 48.12]	15.10	25.09
Ridder	11	4.79	1.94	5.00	[3.65; 5.94]	14.24	19.32
Glubokoe	11	4.21	2.48	3.00	[2.75; 5.68]	7.21	9.82
Altai	10	3.08	1.73	2.80	[2.01; 4.15]	5.78	13.72
Shemonaikha	4	3.12	2.63	3.25	[0.55; 5.70]	6.25	7.50

Note: *n* denotes the number of observation years with available data; SD, standard deviation; 95% CI, the confidence interval for the mean. Kruskal–Wallis test results: SI, H = 22.74, *p* < 0.001; HEF, H = 4.96, *p* = 0.292. Pairwise comparisons for SI (Mann–Whitney test with Bonferroni correction, α = 0.005) showed that Ust-Kamenogorsk differed significantly from Ridder (*p* = 0.001), Glubokoye (*p* = 0.001), and Altai (*p* < 0.001); the difference from Shemonaikha was not statistically significant (*p* = 0.011), partly because of the small sample size (*n* = 5). All other pairwise differences were not significant.

**Table 4:** Mann–Kendall Test Results for API_5_ Trends in the Industrial Settlements of the East Kazakhstan Region, 2014–2024.

Industrial Settlement	S	Z	*p*-Value	Sen’s Slope	Trend	Significance
Ust-Kamenogorsk	−6	−0.396	0.692	−0.150	Decreasing	No (*p* ≥ 0.05)
Ridder	−41	−3.133	0.002	−0.500	Decreasing	Yes (*p* < 0.05)
Glubokoe	−35	−2.719	0.007	−0.300	Decreasing	Yes (*p* < 0.05)
Altai	−33	−2.576	0.010	−0.167	Decreasing	Yes (*p* < 0.05)
Shemonaikha	+6	+0.791	0.429	0.000	Increasing	No (*p* ≥ 0.05)

Note: S denotes the Mann–Kendall statistic; Z, the standardized test statistic; Sen’s slope represents the median change in API_5_ per year; statistical significance was set at *p* < 0.05.

**Table 5:** Spatiotemporal trends in neoplasm morbidity in the East Kazakhstan Region, 2014–2024.

Year	East Kazakhstan Region (Per 100,000 Population in the Respective Group)	Republic of Kazakhstan (Per 100,000 Population in the Respective Group)	Excess Ratio (East Kazakhstan Region/Republic of Kazakhstan)	Rank of the East Kazakhstan Region
2014	1010.0	498.8	2.02	3
2015	1135.1	563.4	2.01	2
2016	1221.6	621.6	1.96	2
2017	1309.0	671.9	1.95	1
2018	1319.5	747.9	1.76	1
2019	1253.3	703.4	1.78	2
2020	789.0	649.8	1.21	7
2021	795.4	725.9	1.10	7
2022	1001.0	735.4	1.36	5
2023	1350.3	829.4	1.63	2
2024	1102.5	800.5	1.38	5

**Table 6:** Lag-Dependent Associations Between Ambient Air Pollution Indicators and Neoplasm Morbidity by Settlement and Age Group (Spearman’s *ρ*, *p* < 0.05).

Region	Age Group	Exposure Indicator (lag)	Spearman’s *ρ*
Ust-Kamenogorsk	Children	SO_2_ (lag 0)	0.79 *
	Adolescents	CO (lag 3)	0.72 *
Ridder	Children	SO_2_ (lag 0)	0.80 *
	Adults	H_2_S (lag 2)	0.70 *
Altai	Adults	SO_2_ (lag 1)	0.87 *
	Children	API_5_ (lag 3)	0.59 *
Glubokoe	Children	API_5_ (lag 1)	0.64 *
	Total population	SO_2_ (lag 0)	0.65 *
Shemonaikha	Adolescents	NO_2_ (lag 1)	1.00 *
	Children	SO_2_ (lag 2)	1.00 *

Note: ρ—Spearman’s rank correlation coefficient; *—statistical significance at *p* < 0.05; lag—time lag (in years). Data for Shemonaikha are provided for reference only; statistical reliability is limited by the small sample size (*n* = 5).

## Data Availability

The original contributions presented in this study are included in the article. Further inquiries can be directed to the corresponding author.
